# Characterization of Multidrug Resistance Patterns of Emerging *Salmonella enterica* Serovar Rissen along the Food Chain in China

**DOI:** 10.3390/antibiotics9100660

**Published:** 2020-09-30

**Authors:** Xuebin Xu, Silpak Biswas, Guimin Gu, Mohammed Elbediwi, Yan Li, Min Yue

**Affiliations:** 1 Department of Microbiology Laboratory, Shanghai Municipal Center for Disease Control and Prevention, Shanghai 200336, China; xbxu@scdc.sh.cn; 2 Institute of Veterinary Sciences & Department of Veterinary Medicine, College of Animal Sciences, Zhejiang University, Hangzhou 310058, China; silpakbiswas@zju.edu.cn (S.B.); m.elbediwi@zju.edu.cn (M.E.); yanli3@zju.edu.cn (Y.L.); 3 Guangxi Institute for Product Quality Inspection, Nanning 530007, China; guguimin@163.com; 4 Animal Health Research Institute, Agriculture Research Centre, Cairo 11435, Egypt; 5 Zhejiang Provincial Key Laboratory of Preventive Veterinary Medicine, Hangzhou 310058, China

**Keywords:** human salmonellosis, *Salmonella* Rissen, multidrug resistance, minimum inhibitory concentration, public health

## Abstract

*Salmonella* spp. are recognized as important foodborne pathogens globally. *Salmonella enterica* serovar Rissen is one of the important *Salmonella* serovars linked with swine products in numerous countries and can transmit to humans by food chain contamination. Worldwide emerging *S*. Rissen is considered as one of the most common pathogens to cause human salmonellosis. The objective of this study was to determine the antimicrobial resistance properties and patterns of *Salmonella* Rissen isolates obtained from humans, animals, animal-derived food products, and the environment in China. Between 2016 and 2019, a total of 311 *S.* Rissen isolates from different provinces or province-level cities in China were included here. Bacterial isolates were characterized by serotyping and antimicrobial susceptibility testing. Minimum inhibitory concentration (MIC) values of 14 clinically relevant antimicrobials were obtained by broth microdilution method. *S*. Rissen isolates from humans were found dominant (67%; 208/311). *S*. Rissen isolates obtained from human patients were mostly found with diarrhea. Other *S*. Rissen isolates were acquired from food (22%; 69/311), animals (8%; 25/311), and the environment (3%; 9/311). Most of the isolates were resistant to tetracycline, trimethoprim-sulfamethoxazole, chloramphenicol, streptomycin, sulfisoxazole, and ampicillin. The *S.* Rissen isolates showed susceptibility against ceftriaxone, ceftiofur, gentamicin, nalidixic acid, ciprofloxacin, and azithromycin. In total, 92% of the *S*. Rissen isolates were multidrug-resistant and ASSuT (27%), ACT (25%), ACSSuT (22%), ACSSuTAmc (11%), and ACSSuTFox (7%) patterns were among the most prevalent antibiotic resistance patterns found in this study. The widespread dissemination of antimicrobial resistance could have emerged from misuse of antimicrobial agents in animal husbandry in China. These findings could be useful for rational antimicrobial usage against *Salmonella* Rissen infections.

## 1. Introduction

*Salmonella* is a gram-negative bacterium that belongs to the *Enterobacteriaceae* family [1]. *Salmonella* spp. are the most important bacterial pathogens among other foodborne pathogens and are responsible for causing gastroenteritis in humans [2]. *Salmonella enterica* subsp. *enterica* includes more than 2600 serotypes and are capable of infecting animals and humans [3,4]. Infections caused by *Salmonella* spp. in farm animals has been documented as the leading cause of considerable economic losses worldwide [5,6].

Nontyphoidal *Salmonella enterica* subsp. *enterica* are responsible for causing significant numbers of food-borne diseases in many countries [1,3,7]. *Salmonella enterica* serovar Rissen (*S*. Rissen) is one of the major *Salmonella* serovars generally found in swine and swine products, chicken meat, and humans with gastrointestinal diseases in different countries [8,9]. The European Food Safety Authority (EFSA) and European Centre for Disease Prevention and Control (ECDC) reported *S.* Rissen as one of the top twenty most common *Salmonella* serovars linked with human infections [7].

The worldwide increase of foodborne infections linked with antimicrobial-resistant pathogenic microorganisms and the dissemination of antimicrobial resistance (AR) is one of key concerns in developing and developed countries [10,11]. On the other hand, another concern for human health in different countries is the emergence of multi-antimicrobial-resistant *Salmonella* strains and the continuous spread of those clones [4,12,13,14]. *Salmonella* spp. are responsible for causing key financial losses in the health care system as well as in the food industries [3].

Incidents of multidrug resistance in *Salmonella* spp., including other bacterial pathogens causing enteric diseases, has been reported in many continents and became a major health issue as this can spread internationally [15,16,17]. The food chain constitutes one of the most important mediums for spreading of antimicrobial resistance [18]. The farm animals are the potential pool of bacterial pathogens harboring multidrug resistance. The utilization of antimicrobials in agriculture for growth promotion of animals and for the treatment of the diseases caused by bacterial pathogens can lead to select antimicrobial-resistant pathogens [3,6]. In different studies, both pig and chicken meats have been documented as the reservoir for drug-resistant *Salmonella* spp. [1,8]. This spread of drug resistance through the food chain is considered as a major public health concern [19,20]. Therefore, an improved surveillance of multidrug resistance and resistance determinants in *Salmonella* is crucial for providing data on the magni tude and spectrum of AR in foodborne pathogens affecting humans and animals in different countries.

Increased AR has been reported in many serovars of *Salmonella* spp. globally [6,21]. However, very limited information on the occurrence of antibiotic resistance of *Salmonella* Rissen is available in China and elsewhere [22]. The objective of the present study was to determine the antimicrobial resistance patterns and properties of 311 *Salmonella* Rissen isolates obtained from humans, animals, animal-derived food products, and the environment from 15 provinces or province-level cities between 2016 and 2019 in China. We also conducted whole genomic sequencing (WGS) to investigate the antimicrobial resistance determinants among the selected MDR isolates.

## 2. Materials and Methods

### 2.1. The Source of Salmonella Isolates 

The Chinese local surveillance system, including over 20 provinces or municipal cities’ CDCs in China, was led by the Shanghai CDC. The overall database has over 50,000 *Salmonella* clinical isolates collected since 2006, when Shanghai CDC joined the Global Foodborne Infections Network under the World Health Organization. During the past decades, in line with local CDCs in mainland China, Shanghai CDC gradually expand to collect *Salmonella* isolates all over China, including samples from humans, animals, food and the environment. The *Salmonella* Rissen isolates and their corresponding metadata were obtained from the Chinese local surveillance system. We selected 311 *S*. Rissen isolates obtained during 2016 to 2019 for this investigation, due to the following reasons: (1) these isolates represent the most recent isolates in the past four years at the time of preparation the manuscript; (2) these isolates were selected to capture the largest regions of mainland China.

### 2.2. Identification of Salmonella Isolates

The isolation of the microorganism was performed based on the protocol suggested by the World Organization for Animal Health Terrestrial Manual [23]. According to this recommendation, isolation of the microorganism was done on xylose lysine deoxycholate agar (XLD agar) plates. Briefly, 25 g of bacterial sample was pre-enriched in buffered peptone water (BPW) at 37 °C overnight. The enriched samples were then inoculated on modified semi-solid Rappaport–Vassiliadis (MSRV) and incubated at 42 °C for 24 h. A loopful of the positive growth taken from the MRSV colony was further inoculated on to xylose lysine deoxycholate (XLD) and was kept in an incubator for overnight. Among the suspected colonies, one colony was seeded in Luria–Bertani (LB) for DNA extraction and validated by polymerase chain reaction (PCR). Distinctive round red colonies with black centers on xylose lysine deoxycholate media were considered as probable *Salmonella* colonies. 

### 2.3. DNA Extraction by Boiling Method and PCR

DNA extraction was done by boiling method. A 1 mL bacterial sample was transferred to a 1.5 mL microcentrifuge tube. The cell suspension was centrifuged for 10 min at 14,000× *g* and the supernatant was discarded. The pellet was resuspended in 300 μL of DNase-RNase-free distilled water by vortexing. The tube was centrifuged at 14,000× *g* for 5 min, and the supernatant was discarded carefully. The pellet was resuspended in 200 μL of DNase-RNase-free distilled water by vortexing. The microcentrifuge tube was incubated for 15 min at 100 °C and immediately chilled on ice. The tube was centrifuged for 5 min at 14,000× *g* at 4 °C. The supernatant was carefully transferred to a new microcentrifuge tube and incubated again for 10 min at 100 °C and chilled immediately on ice. An aliquot of 5 μL of the supernatant was used as the template DNA in the PCR reaction.

### 2.4. PCR Amplification of stn Gene

PCR for *stn* gene, for enterotoxin, was performed to confirm *Salmonella* spp. as recommended previously [24]. Extracted DNA was amplified by PCR using gene specific primers for *stn* forward primer (F1) 5′-TTGTGTCGCTATCACTGGCAACC-3′ and reverse primer (R1) 5′-ATTCGTAACCCGCTCTCGTCC-3′. The PCR protocol for amplification was as follows: initial denaturation at 94 °C for 10 min followed by 35 cycles, (i) denaturation at 94 °C for 45 s; (ii) primer annealing at 58 °C for 45 s, and (iii) primer extension at 72 °C for 45 s followed by final extension at 72 °C for 7 min.

### 2.5. Serotyping by Agglutination Assay

We characterized O and H antigens by agglutination with hyperimmune sera and the serotype of *Salmonella* spp. was identified as per the Kauffmann–White scheme [25].

### 2.6. Antimicrobial Susceptibility Test

Susceptibility to different antimicrobials of all selected isolates was performed as minimum inhibitory concentration (MIC) determinations using a broth microdilution method according to the guidelines of the Clinical and Laboratory Standards Institute (CLSI) [CLSI, 2016]. The broth microdilution method was performed using Muller–Hinton broth and Muller–Hinton agar. In total, 14 clinically relevant antimicrobials from different classes were used to obtain the MIC values. The antimicrobial classes and the MIC range (mg/L) used in this susceptibility assay were penicillin (ampicillin, AMP, 0.125–128), beta-lactams (amoxicillin-clavulanic acid, AMC, 0.5/0.25–64/32), cephems (ceftriaxone, CRO, 0.06–64; cefoxitin, FOX, 0.125–128; ceftiofur, TIO 0.06–64), aminoglycosides (gentamicin, GEN, 0.125–128; streptomycin, STR, 0.125–128), tetracyclines (tetracycline, TET, 0.125–128), quinolones (ciprofloxacin, CIP, 0.03–32; nalidixic acid, NAL, 0.125–128), sulfonamides (trimethoprim/sulfamethoxazole, COT, 0.12/2.38–4/76; sulfisoxazole, FIS, 8–1024), macrolides (azithromycin, AZI, 0.125–128), and phenicols (chloramphenicol, CHL, 0.125–128). The MIC values of the antibiotics used were recorded for all bacterial isolates and compared to the CLSI breakpoints (for ampicillin, amoxicillin–clavulanic acid, ceftriaxone, cefoxitin, gentamicin, streptomycin, tetracycline, ciprofloxacin, nalidixic acid, trimethoprim/sulfamethoxazole, azithromycin, and chloramphenicol) and the breakpoint recommendations from the National Antimicrobial Resistance Monitoring System (NARMS) (for ceftiofur, sulfisoxazole). *Salmonella* Rissen isolates that showed resistant to more than three classes of antimicrobial agents were defined as multidrug-resistant (MDR) isolates.

### 2.7. Genomic Sequencing and Bioinformatic Analysis 

To better investigate the relation between the phenotypic antimicrobial resistance and its genetic determinants, genomic sequencing and bioinformatic analysis were conducted on eight selected extensive multidrug *S*. Rissen isolates among each host, including five from human origin, two from swine origin, and one from chicken meat origin. 

The genomic DNA library was constructed using Nextera XT DNA library construction kit (Illumina, USA, no: FC-131-1024), followed by genomic sequencing using Miseq Reagent Kit v2 300cycle kit (Illumina, USA, No: MS-102-2002). High-throughput genome sequencing was achieved by the Illumina Miseq sequencing platform, as previously described [26,27,28]. The quality of sequencing was checked with FastQC toolkit, while low-quality sequences and joint sequences were removed with trimmomatic [29]. The genome assembly was performed with SPAdes 4.0.1 for genomic scaffolds [30], using the “careful correction” option in order to reduce the number of mismatches in the final assembly with automatically chosen k-mer values by SPAdes. QUAST [31] was used to evaluate the assembled genomes through basic statistics generation, including the total number of contigs and contig length. Multilocus sequence typing (MLST) software (http://www.github.com/tseemann/mlst) was applied for the sequence type of the isolates for the in-house database. Detection of antimicrobial resistance genes was conducted using ABRicate software (http://www.github.com/tseemann/abricate).

### 2.8. Ethical Approval

All procedures performed in studies involving human participants were officially approved by the Shanghai CDC, which was in accordance with the ethical standards of the institutional research committee.

## 3. Results and Discussion

### 3.1. Human Isolates of S. Rissen Are Dominant

**Figure 1:**
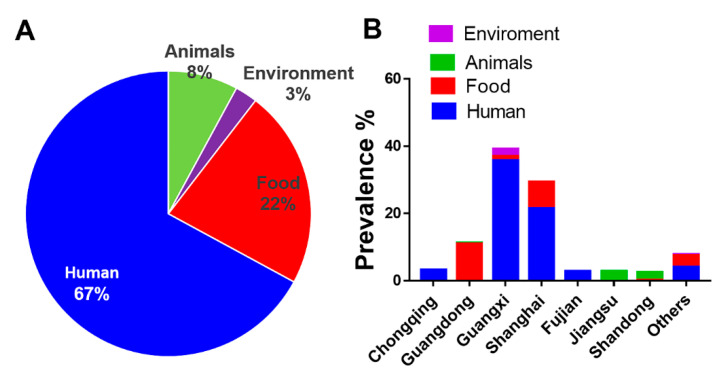
The origin and geographic dynamics of 311 *Salmonella* Rissen isolates examined in this study. (**A**) Prevalence of 311 *S.* Rissen isolates according to the sample sources used in this study. The different sample sources included humans, animals, animal-derived foods, and the environment. (**B**) Prevalence, geographical distribution, and different sources of 311 *S.* Rissen isolates obtained from different provinces or province-level cities in China.

Human salmonellosis is considered as one of the key public health concerns worldwide. In this study, we worked on 311 *S.* Rissen isolates obtained from different sources. We obtained the greatest number of *S.* Rissen isolates from human (67%; 208/311). Out of 208 human samples, we obtained 103 samples from patients with diarrhea, five samples from patients with bacterimia, and 100 samples from asymptomatic carriers. Other *S.* Rissen isolates were obtained from animal-derived foods (22%; 69/311), animals (8%; 25/311), and the environment (3%; 9/311) (Figure 1A). Among 69 samples from animal-derived foods, one sample was obtained from beef, 20 samples were from chicken meat, three samples were from duck meat, and 45 samples were obtained from pork or pig meat. Among 25 samples obtained from animals, 17 samples were obtained from chicken and eight samples were of swine origin.

We found most of the *S.* Rissen isolates from humans were from Guangxi and Shanghai in China (Figure 1B). In a major Salmonella outbreak in the US in 2009, more than 80 people were infected by *S.* Rissen pathogens over four different states of the country [32]. Previous reports demonstrated a number of cases of human infections caused by *S.* Rissen in Demark, Ireland, and UK [33,34]. The risk of salmonellosis in humans as well as the increase of MDR Salmonella clones highlights the importance of the surveillance of rising *S.* Rissen pathogens. It has been found that about 95% of human salmonellosis is linked with the eating of undercooked or contaminated swine meat [35,36,37,38]. *Salmonella* could affect humans at any stages of the food production chain [39,40]. A recent study [41] demonstrated that the Salmonella contamination in animal-derived foods in Guangdong Province in China is very severe, posing significant risk for human infections. Considering the sporadic cases of *Salmonella* Rissen in humans, this study could shed light on the characterization of antibiotic susceptibility profile of *S.* Rissen isolates in humans, causing diarrhea and bacteremia, with the largest number of isolates included to date. This is of clinical significance and could guide regional risk assessments for future outbreaks in China.

### 3.2. S. Rissen Showed Resistant Properties Against Important Antimicrobials

**Figure 2:**
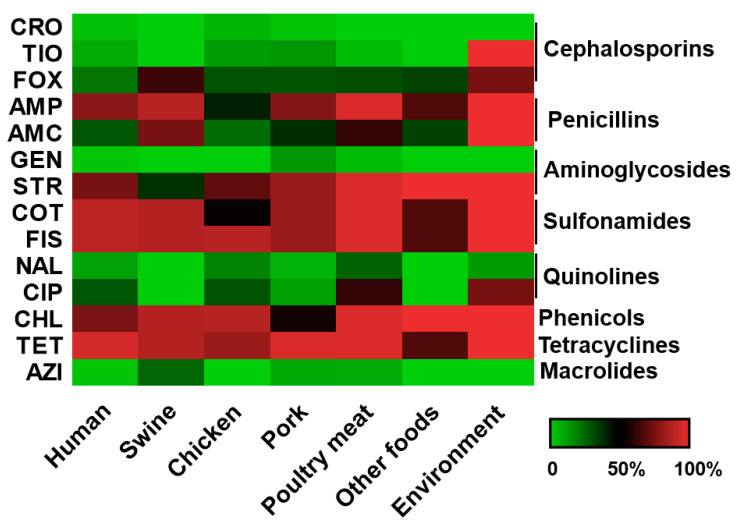
The heatmap of antimicrobial resistance profile for *S*. Rissen isolates according to sample sources based on minimum inhibitory concentration (MIC) values. The 14 antimicrobials used in this study were as follows: ampicillin (AMP), amoxicillin–clavulanic acid (AMC), ceftriaxone (CRO), cefoxitin (FOX), ceftiofur (TIO), gentamicin (GEN), streptomycin (STR), tetracycline (TET), ciprofloxacin (CIP), nalidixic acid (NAL), trimethoprim/sulfamethoxazole (COT), sulfisoxazole (FIS), azithromycin (AZI), and chloramphenicol (CHL). Each cell refers to the percentage of antimicrobial-resistant bacterial isolates recovered from different sample sources with a particular antimicrobial agent, from low (green) to high (red).

We found most of the *S*. Rissen isolates showed resistance to tetracycline, streptomycin, trimethoprim-sulfamethoxazole, chloramphenicol, sulfisoxazole, and ampicillin (Figure 2) which correlates well with other studies and could be linked with the findings that the antimicrobials were commonly used in swine farms in China [42,43]. Tetracycline is one of the most commonly used antimicrobial agents in humans, as well as in veterinary medicine, and is also one of the most extensively used drugs in animal husbandry in China and many other nations. Previous studies from different countries reported a high prevalence of tetracycline resistance in *Salmonella* Rissen [33,44,45]. High resistance to tetracycline could be explained by its extensive use to feed animals and this result was in accordance with other previous studies [46,47]. 

Previous reports described that *Salmonella* isolates displayed resistance against important antibiotics such as tetracycline, streptomycin, ampicillin, chloramphenicol, amoxicillin, neomycin, and sulfonamide [48,49]. Another report [50] demonstrated the widespread occurrence of antibiotic resistance to ampicillin, streptomycin, tetracycline, sulfonamide, and chloramphenicol found in *S*. Rissen isolates from swine farms in upper northern Thailand. Among the *S*. Rissen isolates obtained from pigs in Europe, tetracycline was found to be the most common resistance phenotype [44,49]. A recent study [46] reported that 85.7% of the *S.* Rissen isolates from swine demonstrated resistance to tetracycline in Shandong Province, China. Garcia-Feliz et al. [51] reported 50% of the *S*. Rissen isolates, originating from pigs, were resistant to tetracycline alone. In another study, *S*. Rissen isolates from Thailand were resistant to many antibiotics such as tetracycline, ampicillin, streptomycin, sulfisoxazole, and chloramphenicol [52]. Emerging resistance of *S*. Rissen isolates to clinically relevant antimicrobials are an important public health issue.

### 3.3. High Prevalence of MDR S. Rissen Isolates

**Figure 3:**
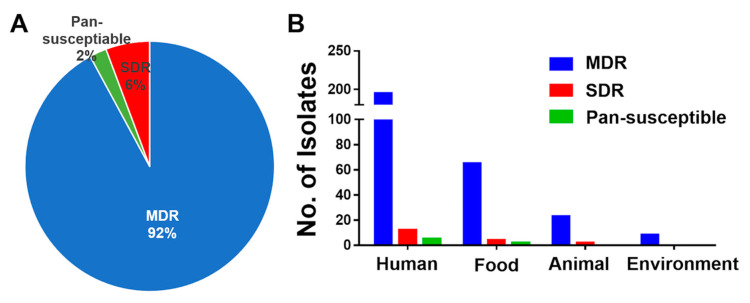
Multidrug-resistant (MDR) properties of 311 *S*. Rissen isolates obtained from humans, animals, animal-derived foods, and the environment in China. (**A**) Percentage of *S*. Rissen isolates found to be MDR in our study. (**B**) MDR *S*. Rissen isolates found from different sources such as humans, food products, animals, and environments. SDR = single-drug resistance, defined as resistance against one type of antimicrobial class.

In total, 92% of the *S*. Rissen isolates were found to be multidrug-resistant (MDR) in our study (Figure 3A). MDR is defined as resistance to three or more different classes of antibiotics. MDR *S*. Rissen isolates were obtained from all sources such as humans, food products, animals, and environments (Figure 3B). *S*. Rissen demonstrating multi-antimicrobial resistance has been recorded in Spain previously, and *S*. Rissen isolates showed MDR properties against four to nine different important drugs [51]. Studies by Tadee et al. [53] in Thailand reported that *S*. Rissen isolates demonstrated resistance against more than three drugs. Previously, Garcıa-Fierro et al. [54] described that 19% of the *S.* Rissen isolates were multidrug-resistant; the isolates were mostly (74%) resistant to tetracycline drug and also demonstrated significant percentages of resistance against ampicillin, streptomycin, sulfonamides, and chloramphenicol, which supports our findings here. 

The high incidence of MDR *Salmonella* Rissen in China found in this study is a serious public health concern. The emergence and dissemination of MDR *Salmonella* are frequently associated with the acquisition of bacterial mobile genetic elements (MGEs) [16,55]. The high occurrence of antibiotic resistance found in this study demonstrated the harmful impact of the unrestricted use of such antibiotics for growth enhancement, as well as in medicine, in China.

### 3.4. Genomic Characterization of an Extensively Drug Resistant Salmonella Rissen

Table 1 described the results of genomic analysis of *S*. Rissen isolates with different antimicrobial resistance genes found in the isolates, which could confer high level of antimicrobial resistance. Genomic analysis of tetracycline-resistant *S*. Rissen isolates showed the presence of *tet* (A) resistance genes responsible for tetracycline resistance. Resistance to tetracycline antimicrobials is controlled by *tet* genes and these genes are generally involved in active efflux of the antimicrobials, as well as in ribosomal protection and enzymatic modification. Among several *tet* genes responsible for tetracycline resistance in *Salmonella*, *tet* genes belong to classes A, B, C, D, and G were found most frequent types of genes [56,57]. *bla*_TEM-1B_ resistance genes were found in ampicillin-resistant *S*. Rissen isolates in this study. The dominant *bla* gene conferring ampicillin resistance in most of the *Salmonella* serovars was found to be different types of *bla*_TEM_ [58,59,60]. Different aminoglycoside resistance genes such as *aadA2, aadA1, aac(6’)-Iaa,* and *aph(3″)-lld* were found here and are demonstrated in Table 1. Among different mechanisms of aminoglycoside resistance, enzymatic modification is the most prevalent in pathogenic bacteria, including *Salmonella* spp. [4,61]. Through genome analysis, we found the *sul3* antibiotic resistance gene in sulfaxisazole-resistant Rissen isolates. This same resistance gene *sul3* was also found in trimethoprim-sulfamethoxazole-resistant isolates. Another important gene *dfrA12* was found in trimethoprim–sulfamethoxazole-resistant *S*. Rissen isolates in our genomic study (Table 1). It has been found in many studies that resistance to sulfonamide antimicrobials is primarily mediated by the *sul1*, *sul2*, and *sul3*genes [62,63]. The major mechanism of trimethoprim resistance is the existence of integron-borne dihydrofolate reductases. The *dfrA12* gene was among the different genes encoding dihydrofolate reductases reported in *Salmonella* previously [64,65,66,67]. The presence of different antimicrobial resistance genes in *S*. Rissen isolates mainly obtained from human demonstrates their MDR properties. 

### 3.5. S. Rissen from Animal and Animal Products with Antimicrobial Resistance

We found 17% of *S*. Rissen isolates came from swine and swine products, 5% of isolates were from chicken, 1% were seafood isolates, and 3% of isolates were from the environment in this study. Pigs are often nonsyndromic carriers of different *Salmonella* serovars [68] and previous studies have shown that swine products could be easily contaminated by *Salmonella* spp. [21,53]. We found *S*. Rissen isolates from animals and animal-originated food products showed resistance against different clinically relevant antibiotics (Figure 2). The dissemination of drug resistance by animal meat products poses a serious public health concern. Previous studies confirmed swine production units in Spain as a main reservoir of *S.* Rissen [49,51]. Hendriksen et al. [33] previously reported that 80% of the *S*. Rissen isolates examined in Denmark were associated with swine products and showed resistance to tetracycline. The study also reported a similar kind of antimicrobial resistance pattern for undercooked as well as and ready-to-eat (RTE) food products found in Thailand. In another research work in South Korea, *Salmonella* Rissen was among the major serovars found in healthy as well as diarrhoeal swine, including a high incidence of resistance to tetracycline, streptomycin, and sulfamethoxazole [9]. Some literatures have reported that *S.* Rissen isolates have also been obtained from other food-producing animals, as well as animal-originated food products, such as poultry and beef, and from human clinical samples, though sometimes with less frequency than other *Salmonella* serovars [45,69,70,71]. This shows the rising of a successful *S*. Rissen clone that can have an effect globally by transmitting to different countries. A very recent study [11] found high levels of resistance among *S*. Rissen isolates recovered from a pig production chain in Thailand and the isolates showed a very high percentage of resistance to ampicillin, tetracycline, and trimethoprim–sulfamethoxazole, and nearly 80% of the bacterial isolates showed a MDR pattern. In another study in the northeastern part of Thailand and Laos, *S*. Rissen isolates showed high frequency of resistance to ampicillin, tetracycline, sulfonamides, and trimethoprim in a swine production unit [72]. These reports showed the significance of stringent monitoring and maintaining of a clean environment in the pork production system. We found very few (3%) *S*. Rissen isolates from the environment and it is interesting to note that sometimes *Salmonella* can survive in the environment for a long time [73,74]. Routine surveillance of pig and poultry farms for *Salmonella* and rapid intervention will significantly improve global food safety and security.

### 3.6. Antimicrobial Susceptibility Pattern of the S. Rissen Isolates

The *S*. Rissen isolates showed susceptibility or low-level resistance against ceftriaxone, ceftiofur, gentamicin, nalidixic acid, ciprofloxacin, and azithromycin (Figure 2). Antibiotic classes, such as fluoroquinolones and beta-lactams, are commonly used in hospitals to treat infections caused by *Salmonella* spp. Quinolones or fluoroquinolones are broad-spectrum antibacterial agents and are used as an important drug of choice for the treatment of the invasive infections in humans and widely used in veterinary medicine. Fluoroquinolone compounds exert their effects by inhibition of some bacterial topoisomerase enzymes, such as DNA gyrase and topoisomerase IV. A small region of *gyrA* was identified as “quinolone resistance-determining region”, or QRDR, and changes in this QRDR region were found in bacterial pathogens with resistance to fluoroquinolones [75]. Beta-lactam antibiotics, which include the cephalosporins class, interfere with the cell wall synthesis by inhibiting the bacterial enzymes. One of the major causes of beta-lactam resistance is by antibiotic-inactivating enzymes known as beta-lactamases [75].

In accordance with our data, some reports in Thailand also demonstrated low-level resistance or susceptibility to ciprofloxacin, ceftriaxone, and ceftiofur. This could be possible because of the restricted use of these antimicrobial agents in the animal-derived food production systems [76,77,78]. Quinolones and third-generation cephalosporins are the antimicrobials most extensively used to treat both human and animal infections. Finding susceptibility to third generation cephalosporins is important, as this group of antibiotics is frequently used against highly invasive bacterial infections. 

### 3.7. ASSuT (Ampicillin, Streptomycin, Sulphonamide, and Tetracycline), and ACSSuT (Ampicillin, Chloramphenicol, Streptomycin, Sulphonamide, and Tetracycline) Pattern of Antimicrobial Resistance

**Figure 4:**
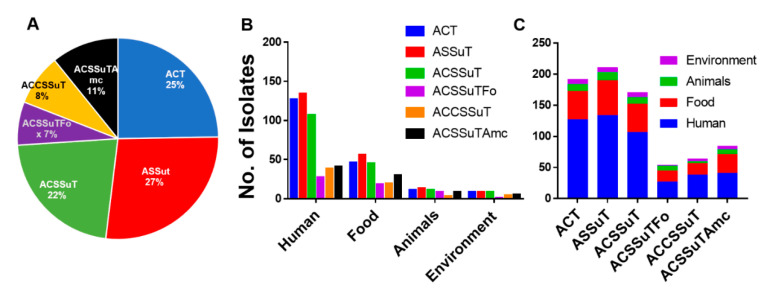
The antimicrobial resistance pattern of *Salmonella* Rissen isolates. (**A**) Pie chart showing percentage of different antimicrobial resistance patterns of all 311 *S*. Rissen isolates obtained from different sources in China. (**B**,**C**) Different antimicrobial resistance patterns of all 311 *S*. Rissen isolates according to sample sources.

For nontyphoidal *Salmonella* or NTS, resistance to five antibiotics, ampicillin, chloramphenicol, streptomycin, sulphonamide, and tetracycline (ACSSuT), is an important resistance pattern. Another important pattern of resistance, ASSuT (ampicillin, streptomycin, sulphonamide, and tetracycline), has also emerged for *Salmonella* species and other foodborne pathogens. *S*. Rissen isolates in this study showed different antibiotic resistance patterns. The occurrence of clinically relevant tetra- or penta-drug resistance patterns such as ASSuT (27%), and ACSSuT (22%) were high, respectively. ACT (ampicillin, chloramphenicol, and tetracycline) (25%), ACSSuTAmc (11%), and ACSSuTFox (7%), were among the other prevalent antibiotic resistance patterns found in this study (Figure 4). ACT (ampicillin, chloramphenicol, and tetracycline), ACSSuTAmc (ampicillin, chloramphenicol, streptomycin, sulfonamides, tetracycline, and amoxicillin-clavulanic acid), and ACSSuTFox (ampicillin, chloramphenicol, streptomycin, sulfonamides, tetracycline, and cefoxitin) antibiotic resistance patterns in *S*. Rissen isolates were mainly obtained from humans and animal-derived foods (Figure 4). Some of these important antimicrobial resistance patterns were also reported in our recent study on *Salmonella* Typhimurium [21]. Figure 4A, with the pie chart, shows the percentage of different antimicrobial resistance patterns of all 311 *S*. Rissen isolates obtained from different sources in China. Figure 4B,C shows different antimicrobial resistance patterns of all 311 *S*. Rissen isolates according to different sample sources.

## 4. Conclusions and Future Perspectives

As a retrospective epidemiological investigation, our study described a high incidence of antimicrobial resistance among *Salmonella* Rissen isolates recovered from diverse sources, especially from humans in China. Our results provide the first outline of rising drug resistance among *S*. *enterica* serovar Rissen, causing humans salmonellosis in China, which is relevant for both food safety and public health. The results we obtained here are more representative of China and could be useful for potential risk evaluation in the future. These findings could signify the possible risk of antimicrobial-resistant *Salmonella* infections in certain provinces or province-level cities in China. Therefore, there must be continuous epidemiological investigations on infections caused by *Salmonella* spp. in humans and animals and more studies are needed to advance our understanding about the development and dissemination of MDR strains. Pigs and swine products are one of the key reservoirs of *Salmonella* Rissen and there is a possibility that enhanced multiple antimicrobial resistance in *S*. Rissen will result in a rising number of human cases. Therefore, further whole genomic sequencing investigations could aim to resolve the genetic diversity in the *S*. Rissen population, as well as the antimicrobial resistance genetic makeup in certain critical antibiotic resistant *S*. Rissen isolates and the potential mechanism for their dissemination. Finding susceptibility to quinolones and third-generation cephalosporins is important and the data presented by this study could be used to recommend suitable therapeutic agents against *Salmonella* Rissen infections in China. Food safety should be improved and uncontrolled use of antimicrobials in growing food-producing animals must be closely monitored to ensure the public health safety.

## Figures and Tables

**Table 1:** Antimicrobial susceptibility and whole genome analysis of MDR *S.* Rissen strains obtained in this study.

	Antibiotic Classes		SAL02425		SAL02454		SAL02475		SAL02482		SAL02490		SAL02560		SAL02592		SAL02603	
			MIC (mg/L)	Related Genes	MIC (mg/L)	Related Genes	MIC (mg/L)	Related Genes	MIC (mg/L)	Related Genes	MIC (mg/L)	Related Genes	MIC (mg/L)	Related Genes	MIC (mg/L)	Related Genes	MIC (mg/L)	Related Genes
**Antimicrobial** **Susceptibility** **testing**	**β-Lactam and β-Lactams inhibitor**	**AMP**	>32	*bla*_TEM-1B_ *blaCTX-M-14*	>32	*bla*_TEM-1B_ *blaCTX-M-14*	>32	*blaCTX-M-14*	>32	*bla*_TEM-1B_ *blaCTX-M-27*	>32	*bla*_TEM-1B_ *blaCTX-M-55*	16		32	*bla*_TEM-1B_	32	*bla*_TEM-1B_
		**AMC**	>32/16		>32/16		>32/16		>32/16		>32/16		8/4		>32/16		>32/16	
	**Amino-** **glycoside**	**STR**	32	*aadA2, aadA1, aac(6′)-Iaa, aph(3* *″)-lld*	64	*aadA2, aadA1, aac(6′)-Iaa*	64	*aadA2, aac(6′)-Iaa*	>64	*aadA2, aadA1, aac(6′)-Iaa*	>64	*aadA2, aadA1, aac(6′)-Iaa, ant(3* *″)-Ia*	>64	*aadA2, aac(6′)-Iaa*	>64	*aadA2, aadA1, aac(6′)-Iaa, ant(3* *″)-Ia*	>64	*aadA2, aadA1, aac(6′)-Iaa*
		**GEN**	>16		>16		2		2		2		1		2		2	
	**Macrolides**	**AZI**	8		8		8		8		8		4		8		8	
	**Quinolone**	**CIP**	0.03		0.03		0.03		0.03		0.06		0.06		0.06		0.06	
		**NAL**	4		4		4		4		4		8		4		4	
	**Phenicol**	**CHL**	8		8		8		8		32	*CmlA2*	32	*CmlA2*	32	*CmlA2*	32	*CmlA2*
	**Sulfaxisazole**	**FIS**	>256	*sul3*	>256	*sul3*	1		>256	*sul3*	>256	*sul3*	1		>256	*sul3*	>256	*sul3*
	**Trimethoprim/Sulphonamide**	**COT**	>32/608	*dfrA12, sul3*	>32/608	*dfrA12, sul3*	>32/608	*dfrA12*	>32/608	*dfrA12, sul3*	>32/608	*dfrA12, sul3*	>32/608	*dfrA12*	>32/608	*dfrA12, sul3*	>32/608	*dfrA12, sul3*
	**Tetra-cyclines**	**TET**	>32	*tet*(A)	>32	*tet*(A)	>32	*tet*(A)	>32	*tet*(A)	>32	*tet*(A)	>32	*tet*(A)	>32	*tet*(A)	>32	*tet*(A)
	**Cephalo-sporines**	**CRO**	>64	*bla*_TEM-1B_ *blaCTX-M-14*	>64	*bla*_TEM-1B_ *blaCTX-M-14*	32	*blaCTX-M-14*	>64	*bla*_TEM-1B_ *blaCTX-M-27*	>64	*bla*_TEM-1B_ *blaCTX-M-55*	2		4	*bla*_TEM-1B_	4	*bla*_TEM-1B_
		**TIO**	>8		>8		>8		>8		>8		1		>8		>8	
		**FOX**	8		16		16		16		16		1		32		32	
**Host**			Human		Human		Human		Human		Human		Live Swine		Chicken meat		Pork	
**Collection place**			Fujian		Shanghai		Fujian		Chongqing		Chongqing		Jiangsu		Guangdong		Guangxi	
**Sequence type**			ST469		ST469		ST469		ST469		ST469		ST469		ST469		ST469	
